# Predicting time to emergency department re-visits and inpatient hospitalization among adolescents who visited an emergency department for psychotic symptoms: a retrospective cohort study

**DOI:** 10.1186/s12888-016-1106-0

**Published:** 2016-11-09

**Authors:** Amir Soleimani, Rhonda J. Rosychuk, Amanda S. Newton

**Affiliations:** 1 Department of Pediatrics, Faculty of Medicine & Dentistry, University of Alberta, Edmonton, AB T6G 1C9 Canada; 2 3-526 Edmonton Clinic Health Academy, 11405 87 Avenue, Edmonton, AB T6G 1C9 Canada

**Keywords:** Adolescent, Psychosis, Emergency department, Pathways to care, Primary care

## Abstract

**Background:**

Adolescents experiencing psychosis may enter the mental health system by a pathway to care that includes or is initiated at the emergency department (ED). However, a better understanding of the pathway to care involving EDs is required to ensure these patients receive the care they require. This study explores physician-based care factors associated with adolescent ED re-visits and inpatient hospitalization following an index ED visit for psychotic symptoms.

**Methods:**

Using administrative data from Alberta, Canada, we identified a cohort of adolescents aged 13–17 years who were discharged after an ED visit for psychotic symptoms between April 1, 2002 and September 29, 2010. Multivariable models estimated times to ED re-visit and inpatient hospitalization for mental health care in a 90-day period after ED discharge.

**Results:**

The cohort was comprised of 208 adolescents. Reduced times to ED re-visit and inpatient hospitalization were associated with: 1) multiple physician visits after discharge (ED re-visit: hazard ratio [HR] 5.93, 95 % confidence interval [CI] 2.09–16.82; inpatient hospitalization: HR 9.43, 95 % CI 1.24–72.00), and 2) post-ED physician care provided in a hospital-based outpatient clinic (ED re-visit: HR 3.07, 95 % CI 1.77–5.29; inpatient hospitalization: HR 3.48, 95 % CI 1.54–7.88). A follow-up visit to a pediatrician, compared to other physician specialties, was associated with earlier inpatient hospitalization (HR 4.45, 95 % CI 1.43–13.87). There was a significant interaction between sex and First Nations status in both models. Females with First Nations status re-visited the ED sooner (HR 3.19; 95 % CI 1.41–7.22) and were hospitalized sooner (HR 4.18; 95 % CI 1.24–14.06).

**Conclusions:**

This study identifies predictors of time to care for adolescents with psychotic symptoms that are worthy of additional investigation. To ensure the pathway to care for these adolescents reduces the duration of untreated problems, health care aspects that require urgent investigation include the type assessments and clinical decisions made during post-ED physician visits.

## Background

Approximately 1 in every 13 adolescents aged 13–18 years will report having experienced a hallucination and/or delusion (psychotic symptoms) [1]. The majority of adults with a lifetime psychotic experience (a collective term for experiences involving psychotic symptoms) report first onset in adolescence or young adulthood [2]. Early detection of psychotic symptoms, but when diagnostic criteria for a psychotic disorder may not be fully met, is currently considered a key strategy to offset, delay or prevent disorders, and reduce years lived with a disability [3]. This effort is based in part on the belief that illness duration influences treatment response and level of recovery [4]. Pathways to care have been studied extensively in this regard [5, 6] with importance placed on reducing the time from onset of psychotic symptoms to the initiation of appropriate psychiatric intervention, also known as the duration of untreated psychosis [5].

Emergency department (ED) visits are regarded as an early contact point in the pathway to care for adolescents before (prodrome) and after a psychotic disorder is diagnosed [7–10]. Recent studies suggest that 3–8 % of pediatric mental health ED presentations in the United States and Canada are for psychotic symptoms [11–14]. ED visits are considered an important contact point to reduce the duration of untreated psychosis through assessment and referral to early intervention programs [5, 15, 16]. Findings from a recent ED-based study indicate, however, that a better understanding of the ED as part of the adolescent pathway to care is needed in order to improve early detection and intervention efforts. In this study, individuals under age 18 years with ED visits for psychotic symptoms had the highest risk of ED re-visit compared to visits for other mental health concerns [17]. Further, the percentage of children with psychosis-related concerns returning to the ED increased substantially over a short time period: 8.7 % at 3 days post-discharge and 15.8 % at 1 month post-discharge [17]. The relationship of the ED re-visits with the timing and type of other health care received (or not received) was not explored in this study although other studies suggest that young adult contact with a primary care physician reduces the likelihood of an ED visit as a first contact [16, 18].

In this study, we retrospectively examined a cohort of adolescents who were discharged from an ED with a psychosis diagnosis to explore the association of physician-based care following an ED visit with time to ED re-visit and inpatient hospitalization for mental health care. Similar investigations have been conducted for other mental health disorders [19, 20]. The goal of this exploratory study was to generate novel hypotheses for prospective investigations aimed at improving pathways to care for adolescents with psychotic symptoms. Informed by published literature on adolescent psychosis [5, 7, 9, 15, 16], we hypothesized that a physician visit for mental health care after an ED visit for mental health care would increase adolescents’ time to ED re-visit and reduce their time to hospital admission. We also hypothesized that more than one physician visit following an ED visit would result in an earlier time to ED re-visit and hospital admission, as this may suggest adolescents are not receiving the care that they require or their conditions are worsening. We made no hypotheses regarding physician type and practice setting as we considered these factors exploratory in our analyses.

## Methods

### Study setting and population

In this study we analyzed data from an 8-year period (April 1, 2002 to September 29, 2010). Data were obtained from the Ambulatory Care Classification System (ACCS), a database of Alberta Health [21] that houses computerized abstracts of all ambulatory care data, including ED data, for the Alberta, Canada. This database contributes to the National Ambulatory Care Records System (NACRS) maintained by the Canadian Institute of Health Information [22], and is subject to processes to ensure data integrity and quality [23]. The Research Ethics Board at the University of Alberta approved the study. The Board assessed all matters required by section 50(1)(a) of the Health Information Act (Alberta, Canada). Subject consent for access to personally identifiable health information was waived as it was deemed to not be reasonable, feasible or practical.

Data were extracted for all adolescents aged 13 to 17 years who were discharged from an ED (104 EDs in total) during the study period with a main ambulatory care diagnosis of psychosis according to the World Health Organization diagnostic codes (International Classification of Diseases 10CA codes F20.x, F21, F22.x, F23.x, F24, F25.x, F28, F29, F30.2) [24]. The main ambulatory diagnosis reflects the main reason for the provision of emergency care, and is coded by trained and supervised medical records nosologists using a uniform protocol [21].

### Cohort creation

A study cohort of 208 adolescents was created with one record per discharged adolescent. If an adolescent had more than one visit concluding in discharge during this time frame, one visit was randomly selected and included. This construction allowed us to address time to follow-up visits after a specific ED visit and removed any requirement to adjust for subject-specific correlation in the analyses.

### Study variables

#### ED visit characteristics

We used the ACCS to identify psychiatric comorbidity at the ED index visit using diagnostic codes assigned in the secondary and tertiary diagnostic fields (International Classification of Diseases 10CA codes F10.x-F19.x, F30.x-F34.x, F38.x, F40.x-F45.x, F48.x, F50.x, F55, F59, F60.x-F64.x, F68.x-F69, F90.x-F95.x, F99) [24]. We also identified the triage level for ED visits, which was assigned according to the Canadian Triage and Acuity Scale [25, 26], a five-level ordinal scale (1: resuscitation, 2: emergent, 3: urgent, 4: semi-urgent, 5: non-urgent). The scale has good validity and inter-rater reliability and allows clinicians to triage patients according to the type and severity/acuity of the presenting signs and symptoms.

#### Adolescent sociodemographics

ACCS data were linked to a registry file to identify age and sex at the time of the index ED visit, and categorize adolescents using a socioeconomic status proxy. Details of this proxy have been published elsewhere [19]. The four groups adolescents were allocated to were as follows: 1) human services program recipient (an adolescent whose family received income support and health benefits from the government of Alberta), 2) government sponsored program subsidy recipient (an adolescent whose family received partial or full subsidies, or disability benefits from the government), 3) no subsidy support (an adolescent whose family received no support from the government), and 4) First Nations status (the adolescent who was registered under the federal Indian Act).

### Health care utilization variables

ACCS data provided dates and diagnostic codes for ED visits after the index visit, which allowed us to identify ED re-visits for mental health care. Linkage to the Canadian Institute for Health Information Discharge Abstract Database [27] provided the start and end dates of hospital stays to identify inpatient hospitalization for mental health care. Linkage to a physician claims file provided: 1) the number of physician visits prior to and following the index visit, 2) up to three diagnostic codes for each visit, which allowed us to distinguish between follow-up visits that were mental health related and those that were not, 3) the type of practicing physician (general practitioner [GP], pediatrician, psychiatrist, other), and 4) the type of facility where the physician provided care (practitioner’s office, community-based mental health facility, hospital-based outpatient clinic, other).

### Main outcome measures

Within a 90-day period following the index ED visit, the outcomes were: 1) time to ED re-visit for mental health care (time from the index visit end date to the first subsequent ED visit) and 2) time to inpatient hospitalization for mental health care (time from the index ED visit end date to the first subsequent ED visit that had a disposition of hospital admission).

### Statistical analysis

We developed multivariable Cox proportional hazards models to investigate the effect of predictor variables on time to ED re-visit and time to inpatient hospitalization. We included in the models physician-based variables to explore their effect on the main outcomes: the number of physician visits in a 30-day post-discharge period, the type of follow-up visit (mental health related, medical related), the type of physician seen at follow-up (GP, pediatrician, psychiatrist, other), and the type of facility where the physician provided care (practitioner’s office, community-based mental health facility, hospital-based outpatient clinic, other). We adjusted for comorbidity [16] and triage level [6, 17, 28] at the index ED visit, as well as for physician visits for mental health care 30 days prior to the ED visit [16]. While evidence is mixed on their effect on health services utilization in our study population, we also adjusted for age and sex, and socioeconomic status as some studies have reported a significant impact on outcomes [5, 16, 28]. In the first model, all categorical variables were entered separately using indicator variables (e.g., socioeconomic status had three indicator variables for the categories First Nations status, human services program recipient, and government sponsored program recipient, and the reference category was no subsidy). Variables and interaction terms (age × sex, age × socioeconomic status, sex × socioeconomic status) were entered into each of the full models and removed, one at a time, if not statistically significant (*p* > 0.05) to obtain the final models. In particular, if an indicator variable was not statistically significant, the category was combined with the reference category because there was no evidence that the two categories should be separate. The final models were assessed by tests of proportional hazards assumptions and deviance residual diagnostics. The final models met proportional hazards assumptions. Adjusted hazard ratios (HR) and 95 % confidence intervals (CI) are reported.

## Results

### Cohort description

Table 1 presents adolescent sociodemographics and clinical features of index ED visits made by the 208 adolescents in the study cohort. Psychiatric co-morbidity was indicated for 50 adolescents at the index ED visit; the most common co-morbidities were anxiety disorder (28.0 %), mood disorder (26.0 %), and mental or behavioural disorder secondary to substance abuse (24.0 %). In the 90-day post-discharge period, 37 adolescents had no physician follow-up visit, while 31 adolescents had a single visit and 140 adolescents had multiple visits.

**Table 1:** Sociodemographics and clinical features at the ED index visit among adolescents discharged following a discharge diagnosis of psychosis (*n* = 208). Figures are number, percentage unless otherwise stated

	Number	Percent
Age at ED visit, mean (SD)	14.95 (1.35)	
Sex		
female	92	44.23 %
male	116	55.77 %
Socioeconomic status proxy		
First Nations status	27	12.98 %
no subsidy	116	55.77 %
human services program recipient	19	9.13 %
government sponsored program recipient	46	22.12 %
Main ambulatory diagnosis		
Schizophrenia, schizotypal disorder (ICD F20.0-F20.9, F21)	46	22.12 %
Persistent delusional disorder (ICD F22.0, F22.8, F22.9)	11	5.29 %
Acute and transient psychotic disorder (ICD F23.0-F23.3, F23.8-F23.9)	33	15.87 %
Schizoaffective disorder, mania with psychotic symptoms (ICD F25.0-F25.2, F25.8-F25.9, F30.2)	7	3.37 %
Nonorganic psychotic disorder (ICD F28)	8	3.85 %
Unspecified nonorganic psychotic disorder (ICD F29)	103	49.52 %
Psychiatric comorbidity		
Yes	50	24.00 %
No	158	76.00 %
Acuity assigned to self-harm using triage level		
1 (resuscitation)	4	1.92 %
2 (emergent)	20	9.62 %
3 (urgent)	80	38.46 %
4 (semi-urgent)	56	26.92 %
5 (non-urgent)	12	5.77 %
Unknown^a^	36	17.31 %

Legend: *ICD* International Classification of Diseases, *SD* standard deviation; ^a^Triage level was a mandatory reportable field for EDs by April 1, 2006

### Physician-based care features associated with time to ED re-visit

In a 90-day post-discharge period, compared to adolescents who had no physician visit or a single visit, adolescents with multiple physician visits had a reduced time to ED re-visit (Table 2; HR 5.93; 95 % CI, 2.09–16.82). A reduced time to ED re-visit was also associated with physician follow-up visits that took place in hospital-based outpatient settings (HR 3.07; 95 % CI, 1.77–5.29). The type of physician seen in the post-discharge period did not predict time to ED re-visit. There was a significant interaction between sex and First Nations status in this model. Females with First Nations status had a reduced time to ED return (HR 3.19; 95 % CI, 1.41–7.22).

**Table 2:** Adjusted hazard ratios and 95 % confidence intervals for predictors of ED re-visit for mental health care within 90 days of the index ED visit

	Number	Adjusted hazard ratio	95 % confidence interval
Sex × socioeconomic status proxy (interaction)			
Male, First Nations status vs. no subsidy/government sponsored program recipient/human services program recipient	17 vs. 99	0.84	0.32 to 2.17
Female, First Nations status vs. no subsidy/government sponsored program recipient/human services program recipient	10 vs. 82	3.19	1.41 to 7.22
Number of physician follow-up visits for any reason within 30 days following index ED visit			
0 or 1	68	1.00	Reference
Greater than 1	140	5.93	2.09 to 16.82
Facility type where follow-up visit occurred			
Hospital-based outpatient care	83	3.07	1.77 to 5.29
No visit/community mental health facility/practitioner’s office/other	125	1.00	Reference

### Physician-based care features associated with time to inpatient hospitalization

Multiple physician follow-up visits made by children (HR 9.43; 95 % CI, 1.24–72.00) and type of physician seen at follow-up (pediatrician; HR 4.45; 95 % CI, 1.43–13.87) predicted a decreased time to inpatient hospitalization within 90 days of the index ED visit (Table 3). A reduced time to inpatient hospitalization was also associated with physician follow-up visits that took place in hospital-based outpatient settings (HR 3.48; 95 % CI, 1.54–7.88). Similar to time to ED re-visit, females with First Nations status predicted sooner inpatient hospitalization (HR 4.18; 95 % CI, 1.24–14.06).

**Table 3:** Adjusted hazard ratios and 95 % confidence interval for predictors of inpatient hospitalization for mental health care within 90 days of the index ED visit

	Number	Adjusted hazard ratio	95 % confidence interval
Sex × socioeconomic status proxy (interaction)			
Male, First Nations status vs. no subsidy/government sponsored program recipient/human services program recipient	17 vs. 99	0.57	0.13 to 2.49
Female, First Nations status vs. no subsidy/government sponsored program recipient/human services program recipient	10 vs. 82	4.18	1.24 to 14.06
Number of physician follow-up visits for any reason within 30 days following index ED visit			
0 or 1	68	1.00	Reference
Greater than 1	140	9.43	1.24 to 72.00
Physician type at follow-up visit			
Pediatrician	8	4.45	1.43 to 13.87
No visit/general practitioner/psychiatrist/other	200	1.00	Reference
Facility type where follow-up visit occurred			
Hospital-based outpatient care	83	3.48	1.54 to 7.88
No visit/community mental health facility/practitioner’s office/other	125	1.00	Reference

## Discussion

Understanding of factors that influence pathways to care for psychosis is regarded as crucial to improving treatment access [5]. Several novel results from this exploratory study can be used to inform future prospective studies of pathways to care for adolescents with psychotic symptoms. Consistent with our hypotheses, adolescents with multiple physician visits following an ED visit for psychosis returned to the ED significantly sooner, and were hospitalized sooner than adolescents with one or no post-ED physician visits. This finding provides a new perspective to consider for adolescent pathways to care. While a number of other studies have examined the types of contacts and referral sources on pathways to care [5], the relationship between physician contact, ED use and inpatient hospitalization was not statistically modelled. However, whether our study’s findings point to a group of adolescents who remain in a psychiatrically unstable condition despite repeated physician-based care, or whether, despite physician-based care, adolescents’ conditions worsened, could not be determined by this study. Future prospective investigations of how: 1) the adolescents’ psychopathological states, 2) physician decision processes during different patient contacts, 3) the types of assessment and clinical decisions that characterize follow-up visits to physicians, 4) adolescent contact with other professionals (e.g., law enforcement, school counsellors), and 5) the availability of inpatient beds at the time of an ED visit affect pathways to care, are critical to understanding use of EDs and inpatient hospitalization for psychosis-related health care and time to these events. Other studies support the need for these investigations as well. Pathways to care for psychosis are described as complex [5]. Negative experiences with treatment have been shown to contribute to delays in future help seeking [29] and an insidious mode of onset (psychotic symptoms appearing incrementally) has been associated with longer duration of untreated psychosis compared to acute onset [30]. Other research also suggests that adolescent care pathways (including type of care and time to care) are influenced and directed by parents and/or guardians [9].

In this study, mental health follow-up visits to physicians after the ED visit were not significant in the models; that is, such visits did not affect adolescent time to ED re-visit or inpatient hospitalization. It may be that adolescents and their parents are presenting with subtle and negative symptoms of psychosis rather than positive ones. We did not have the necessary patient-level data, however, to study this. A recent international survey of GPs found that while most have a strong understanding of positive symptoms, there was a lack of understanding among many GPs of the subtle symptoms of psychosis such as sleep disturbances, aggression, and social withdrawal [31]. Further investigation as to whether this lack of understanding also applies to adolescents is warranted. While research has advocated for the use of ‘upstream’ health care such as physician-based primary care compared to more ‘downstream’ care pathways such as the ED, upstream health care services must be able to detect early onset illnesses and reduce the duration of untreated psychosis [16, 18, 32].

Although we had no a priori hypotheses regarding the role of physician type and practice setting in time to ED re-visit or inpatient hospitalization, both were significant in our models. Follow-up visits to a pediatrician within 90 days of the index ED visit resulted in significantly shorter times to inpatient hospitalization. Future studies should consider exploring whether pediatricians are able to better differentiate than other physicians between negative and subtle symptoms of early psychosis and typical developmental milestones, thus, determine which adolescents require intensive assessment and treatment. That follow-up visits occurring in hospital settings were associated with a shorter time to ED return and hospitalization suggests further investigation of whether the physical proximity of a follow-up visit (i.e., hospital-based physician care in the same physical location as the ED) or physician behaviour (such as sending an adolescent to the ED during a follow-up visit) are associated with ED use.

In our study we also found that female adolescents with First Nations status had shorter time to ED re-visit and inpatient hospitalization. In general, these findings complement previous research, which has shown that First Nations children were at a higher risk of returning to the ED for mental health concerns and that this risk increased with age [20]. To date, the mental health of First Nations adolescents, particularly those with early onset psychosis, is considerably under-studied. We are not aware of any studies examining gender differences in health care utilization. The most current documentation of the mental health of First Nations adolescents in Canada has focused on death by suicide, as well as alcohol and other drug use [33, 34]. Health care utilization has not been studied. Our findings suggest that ED and physician-based care of First Nations adolescents with psychosis requires significantly more attention including study of gender differences in health care utilization, and further understanding cultural interpretations of the positive and negative symptoms of psychosis.

Several limitations should be considered in interpreting our findings. First, while our work stands as an exploratory analysis, more research is required to further understand the predictors of time to ED re-visit and inpatient hospitalization for adolescents. Some potential explanatory variables were not available from our data source, the ACCS, such as data for visits to non-physician services (e.g., mental health professionals in the community) that can occur parallel to physician-based visits [35], the adolescent’s support system, and encounters with law enforcement. These variables could influence: 1) the number of visits made to a physician following an index ED visit, and 2) ED visits (as ED visits may follow dangerous or unusual behaviours or a critical incident for the young person necessitating intervention from family, friends, and/or law enforcement). Prospectively collecting these variables and adding them to future statistical models would provide a more comprehensive risk model, and help to explore pathways to care for psychosis in adolescence from new perspectives. Second, the relatively few events and heavy censoring in our models resulted in very large confidence intervals for several of the findings, and these results should be interpreted cautiously. The results of study are considered exploratory, however, and we feel they still provide important considerations for future studies. Finally, the databases used do not identify all Aboriginal adolescents; non-Treaty Status, Inuit and Métis adolescents were not included. While not the main focus of our study, this sociodemographic was a significant predictor in our models. Further examination of health care utilization for non-Treaty Status, Inuit and Métis adolescents is warranted.

## Conclusions

This exploratory study has identified predictors of time to ED re-visit and inpatient hospitalization among adolescents who visited an ED for psychotic symptoms. Study findings can be used to inform additional investigations of pathways to care. Additional investigations should include studies of how adolescents’ psychopathological states; care provided during post-emergency, follow-up visits by physicians; physician competencies and skills; contact with other professionals (e.g., law enforcement, school counsellors); and support systems (e.g., family members) affect ED use and inpatient hospitalization.

## Abbreviations

ACCS: Ambulatory care classification system
CI: Confidence interval
ED: Emergency department
GP: General practitioner
HR: Hazard ratio

## References

[1] Kelleher I, Connor D, Clarke M, Devlin N, Harley M, Cannon M (2012). Prevalence of psychotic symptoms in childhood and adolescence: a systematic review and meta-analysis of population-based studies. Psychol Med.

[2] McGrath JJ, Saha S, Al-Hamzawi AO, Alonso J, Andrade L, Borges G (2016). Age of onset and lifetime projected risk of psychotic experiences: cross-national data from the World Mental Health Survey. Schizophr Bull.

[3] McGorry PD, Killackey EJ (2002). Early intervention in psychosis: a new evidence based paradigm. Epidemiol Psychiatr Soc.

[4] Perkins D, Hongbin GU, Boteva K, Lieberman J (2005). Relationship between duration of untreated psychosis and outcome in first-episode schizophrenia: a critical review and meta-analysis. Am J Psychiatry.

[5] Anderson K, Fuhrer R, Malla A (2010). The pathways to mental health care of first-episode psychosis patients: a systematic review. Psychol Med.

[6] Singh S, Grange T (2006). Measuring pathways to care in first-episode psychosis: a systematic review. Schizophr Res.

[7] Addington J, van Mastrigt S, Hutchinson J, Addington D (2002). Pathways to care: help seeking behaviour in first episode psychosis. Acta Psychiatr Scand.

[8] Cheung D, Roper L, Purdon SE (2014). Pathways to (specialized) care: patient costs and contacts en route to a first-episode psychosis clinic. Early Interv Psychiatry.

[9] O’Callaghan E, Turner N, Renwick L, Jackson D, Sutton M, Foley S (2010). First episode psychosis and the trail to secondary care: help-seeking and health-system delays. Soc Psychiatry Psychiatr Epidemiol.

[10] Platz C, Umbricht D, Cattapan-Ludewig K, Dvorsky D, Arbach D, Brenner H (2006). Help-seeking pathways in early psychosis. Soc Psychiatry Psychiatr Epidemiol.

[11] Sheridan DC, Spiro DM, Fu R, Johnson KP, Sheridan JS, Oue AA (2015). Mental health utilization in a pediatric emergency department. Pediatr Emerg Care.

[12] Newton AS, Ali S, Johnson DW, Haines C, Rosychuk RJ, Keaschuk RA (2009). A 4-year review of pediatric mental health emergencies in Alberta. CJEM.

[13] Cappelli M, Glennie JE, Cloutier P, Kennedy A, Vloet M, Newton A (2012). Physician management of pediatric mental health patients in the emergency department: assessment, charting, and disposition. Pediatr Emerg Care.

[14] Pittsenbarger ZE, Mannix R (2014). Trends in pediatric visits to the emergency department for psychiatric illnesses. Acad Emerg Med.

[15] Pira S, Durr G, Pawliuk N, Joober R, Malla A (2013). Mode of entry to an early intervention service for psychotic disorders: determinants and impact on outcome. Psychiatr Serv.

[16] Anderson KK, Fuhrer R, Schmitz N, Malla AK (2013). Determinants of negative pathways to care and their impact on service disengagement in first-episode psychosis. Soc Psychiatry Psychiatr Epidemiol.

[17] Newton AS, Ali S, Johnson DW, Haines C, Rosychuk RJ, Keaschuk RA (2010). Who comes back? Characteristics and predictors of return to emergency department services for pediatric mental health care. Acad Emerg Med.

[18] Anderson KK, Fuhrer R, Wynant W, Abrahamowicz M, Buckeridge D, Malla A (2013). Patterns of health services use prior to a first diagnosis of psychosis: the importance of primary care. Soc Psychiatry Psychiatr Epidemiol.

[19] Newton AS, Rosychuk RJ, Niu X, Radomski AD, McGrath PJ. Emergency department use and postvisit care for anxiety and stress disorders among children: a population-based cohort study in Alberta, Canada. Pediatr Emerg Care. 2016;32:658-63. 10.1097/PEC.0000000000000747 PMC5068196 26945191

[20] Newton AS, Rosychuk RJ, Dong K, Curran J, Slomp M, McGrath PJ (2012). Emergency health care use and follow-up among sociodemographic groups of children who visit emergency departments for mental health crises. CMAJ.

[21] Alberta Health and Wellness. Ambulatory Care in Alberta using Ambulatory Care Classification System Data. Edmonton: Government of Alberta; 2004.

[22] Canadian Institute for Health Information. National Ambulatory Care Reporting System (NACRS) Metadata. 2015. https://www.cihi.ca/en/types-of-care/hospital-care/emergency-and-ambulatory-care/nacrs-metadata. Accessed 3 Aug 2016.

[23] Canadian Institute for Health Information. Data Quality Documentation, National Ambulatory Care Reporting System – Multi-Year Information. 2015. https://www.cihi.ca/sites/default/files/document/nacrs_multi-year_info_en.pdf. Accessed 3 Aug 2016.

[24] World Health Organization. International Statistical Classification of Diseases and Related Health Problems. 2010. http://apps.who.int/classifications/icd10/browse/2010/en. Accessed 6 May 2011.

[25] Gravel J, Manzano S, Arsenault M (2009). Validity of the Canadian Paediatric Triage and Acuity Scale in a tertiary care hospital. CJEM.

[26] Warren DW, Jarvis A, LeBlanc L, Gravel J (2008). CTAS National Working Group. Revisions to the Canadian Triage and Acuity Scale Paediatric Guidelines (PaedCTAS). CJEM.

[27] Canadian Institute for Health Information. Discharge Abstract Database (DAD) Metadata. https://www.cihi.ca/en/types-of-care/hospital-care/acute-care/dad-metadata. Accessed 23 Aug 2016.

[28] Perkins DO, Gu H, Boteva K, Lieberman JA (2005). Relationship between duration of untreated psychosis and outcome in first-episode schizophrenia: a critical review and meta-analysis. Am J Psychiatry.

[29] Monteiro VB, dos Santos JQ, Martin D. Patients’ relatives delayed help seeking after a first psychotic episode. Rev Bras Psiquiatr. 2006;28(2):104–10. 10.1590/s1516-44462006000200006 16810392

[30] Morgan C, Abdul-Al R, Lappin JM, Jones P, Fearon P, Leese M, Croudace T, Morgan K, Dazzan P, Craig T, Leff J, Murray R, AESOP Study Group. Clinical and social determinants of duration of untreated psychosis in the AESOP first-episode psychosis study. Br J Psychiatry. 2006;189:446-52. 10.1192/bjp.bp.106.021303 17077436

[31] Simon AE, Lester H, Tait L, Stip E, Roy P, Conrad G, Hunt J, Epstein I, Larsen TK, Amminger P, Holub D, Wenigová B, Turner M, Berger GE, O’Donnell C, Umbricht D. The International Study on General Practitioners and Early Psychosis (IGPS). Schizophr Res. 2009;108(1-3):182-90. 10.1016/j.schres.2008.11.004 19087897

[32] Malla AK, Norman RMG, Voruganti LP Improving outcomes in schizophrenia: the case for early intervention. CMAJ. 1999;160:843-6. PMC1230170 10189434

[33] Gfellner BM, Hundleby JD. Patterns of drug use among Native and white adolescents: 1990-1993. Can J Public Health. 1995;86(2):95-7. 7757900

[34] Alaghehbandan R, Sikdar KC, MacDonald D, Collins KD, Rossignol AM. Unintentional injuries among children and adolescents in Aboriginal and non-Aboriginal communities, newfoundland and labrador, Canada. Int J Circumpolar Health. 2010;69(1). 10.3402/ijch.v69i1.17386 20167157

[35] Lincoln C, Harrigan S, McGorry PD. Understanding the topography of the early psychosis pathways. An opportunity to reduce delays in treatment. Br J Psychiatry. 1998;172:21-25. 9764122

